# Applying Small-Scale DNA Signatures as an Aid in Assembling Soybean Chromosome Sequences

**DOI:** 10.1155/2010/976792

**Published:** 2010-08-19

**Authors:** Myron Peto, David M. Grant, Randy C. Shoemaker, Steven B. Cannon

**Affiliations:** USDA-ARS-CICGR Unit and Department of Agronomy, Iowa State University, Ames, IA 50011, USA

## Abstract

Previous work has established a genomic signature based on relative counts of the 16 possible dinucleotides. Until now, it has been generally accepted that the dinucleotide signature is characteristic of a genome and is relatively homogeneous across a genome. However, we found some local regions of the soybean genome with a signature differing widely from that of the rest of the genome. Those regions were mostly centromeric and pericentromeric, and enriched for repetitive sequences. We found that DNA binding energy also presented large-scale patterns across soybean chromosomes. These two patterns were helpful during assembly and quality control of soybean whole genome shotgun scaffold sequences into chromosome pseudomolecules.

## 1. Introduction

The soybean (*Glycine max* (L.) Merr.) genome sequencing project was conducted using the whole genome shotgun strategy [1], with the DOE's Joint Genome Institute producing sequence and primary assemblies, and NSF and USDA funded groups providing genetic and physical map resources to integrate the genome into chromosome-scale assemblies [2]. In a whole genome shotgun (WGS) strategy, overlapping paired-end reads are assembled into scaffolds on the basis of sequence overlaps and clone-size information. More than five thousand sequence-based markers were used in the soybean genome assembly to help order and orient scaffolds [3, 4]. Despite the large number of available markers, a significant hurdle in the assembly of scaffolds into pseudomolecules is that genetic markers give poor resolution in the centromeric and pericentromeric regions due to the lack of recombination events [5, 6]. We present two techniques, dinucleotide signature and binding energy, which were useful in assessing the soybean chromosome assemblies and may be of use for other genome assembly projects.

## 2. Results

**Figure 1:**
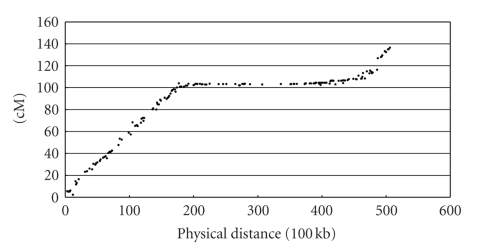
Physical (horizontal) versus genetic (vertical) distance for soybean chromosome 6. Note the flat region in the middle of the chromosome, corresponding to a portion of the chromosome with few recombination events. This hinders accurate marker-based placement of scaffolds in that region. The genetic distances are taken from the Soybean Consensus Map 4.0 [3, 4].

A WGS sequencing project is often divided into two phases: (1) assembly of the reads into scaffolds based on sequence overlap and (2) construction of chromosome pseudomolecules by placing and orienting the scaffolds using other information (i.e., genetic and physical maps). We found that the genetic map, while generally collinear with the genomic sequence, showed widely varying rates of recombination. Figure 1 shows chromosome 6 of soybean (formerly linkage group C2), which illustrates the phenomenon. The horizontal section in the middle of the chromosome covers the centromeric and pericentromeric regions where a large physical distance corresponds to a small genetic distance. The relative lack of recombination in this region results in poor resolution and difficulties in ordering and orienting those scaffolds. In contrast, the euchromatic regions at either end display high genetic-to-physical ratios (in the range of 1 centimorgan per 200,000 bases [1 cM/200 kb]), enabling confident placement of most scaffolds. We were able to use chromosomal-scale signals in both dinucleotide signature differences and binding energies as an aid in ordering and orienting scaffolds in the soybean genome. 

**Figure 2:**
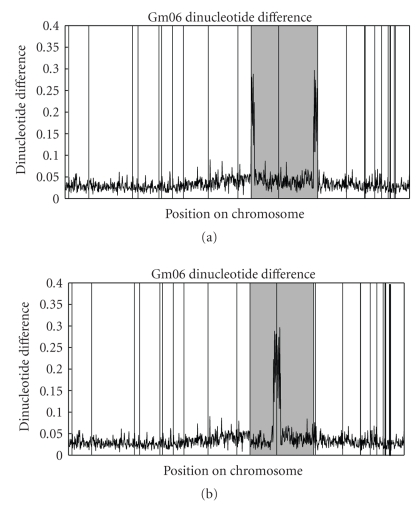
The plots in (a) and (b) show the dinucleotide difference of two assemblies of chromosome 6. The vertical lines correspond to scaffold boundaries. The dinucleotide signature of the darkened two scaffolds provided information about their orientation. Note the peaks at the edges of those two scaffolds. In (b) the orientation of both scaffolds has been reversed in order to unify the assumed centromere. (Other scaffold-order changes outside of the shaded areas were made on the basis of other information, including marker and synteny analyses).

Plots of binding energy and dinucleotide differences were overlaid with scaffold boundaries. Figure 2 gives an example of a dinucleotide plot along with the scaffold boundaries for chromosome 6. The darkened scaffolds show peaks that we believe correspond to the centromere, based on concentrated arrays of 91-92 base satellite repeats [5, 6] at those locations. Gradients shown in Figure 2, as well as other supporting information (below) led to the chromosomal build shown in Figure 2(b).

The highlighted scaffolds in Figure 2 are scaffold_58 and scaffold_35 (from the Arachne build preceding the Glyma1.01 assembly release [1]). Scaffold_58 contains 14 mapped markers, ranging from 102.9 to 103.9 cM on the 4.0 consensus genetic map [4], and tentatively indicating that this scaffold should have a positive orientation. Scaffold_35 contains 7 mapped markers, ranging from 103.1 to 103.5 cM, also tentatively indicating a positive orientation. The scaffolds were initially placed with scaffold_58 first (cM 103.37), then scaffold_35 (103.41) in the orientations mentioned. These cM values are below the resolution of the map, however, so are fair game for re-evaluation. The dinucleotide plot, with peaks at the edges of both scaffolds, suggested that a reversal of orientation of both scaffolds was appropriate. This change was also supported by two FPC contigs [7] that span the boundaries between scaffolds.

The contig WmFPC_Contig240 spans the boundary between scaffold_35 and scaffold_882, the scaffold directly to the right of scaffold_35 (see the soybase genome browser at http://soybase.org/). WmFPC_Contig240 also spans the boundary between scaffold_882 and scaffold_3195, the scaffold directly to the right of scaffold_882. This strongly suggests that the position and orientation of scaffold_35 shown in Figure 2(b) is indeed correct. WmFPC_Contig6136 spans the boundary of scaffold_58 and scaffold_35 across the centromere. Integrity of this centromere-spanning scaffold is suspect (Will Nelson, personal communication), but together with the evidence above, the physical map provides some supporting evidence of both the correct orientation of scaffold_35 and scaffold_58.

**Figure 3:**
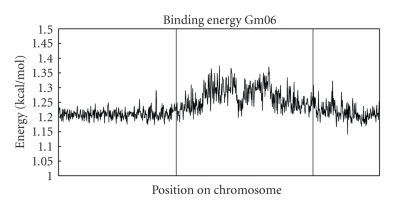
Binding energy versus chromosomal location for soybean chromosome 6. The two vertical lines correspond to boundaries between euchromatic and heterochromatic regions, as determined from Figure 1.

 Plots of dinucleotide binding energy along the chromosome versus genetic position were similarly useful in pseudomolecule assembly. We calculated the binding energy of 50 kb segments by adding up the energy of all the individual dinucleotides and averaging by the total count. When we plotted the averages across a whole chromosome, we observed large-scale patterns. The binding energy and variability tended to increase in the centromeric and pericentromeric regions. The average and standard deviation of binding energy from the beginning 17.5 million bases (Mb) and last 10 Mb of DNA from chromosome 6 (delineated by vertical lines in Figure 1) were 1.21 and 0.02. Figure 3 shows a plot of binding energy with vertical lines separating the regions. The average and standard deviation of the binding energy from the remaining middle section of the chromosome were 1.27 and 0.04. In addition to a larger variation, there tended to be large-scale oscillations present in the middle pericentromeric and centromeric regions of chromosomes. It was those large-scale patterns that we were able to exploit in assembling and orientating of scaffolds in a manner similar to our use of the dinucleotide signature.

**Figure 4:**
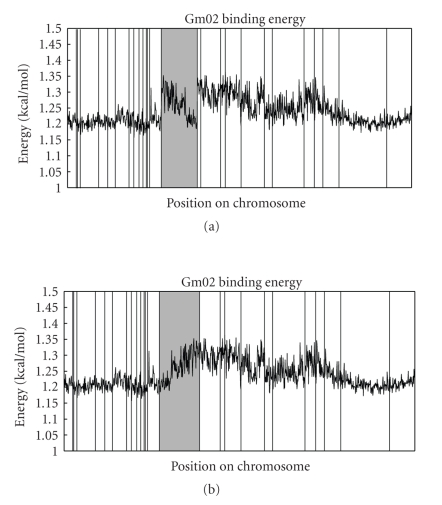
The plots in (a) and (b) show the binding energy of 50 kb segments of two assemblies of chromosome 2. The vertical lines again correspond to scaffold boundaries. The darkened scaffold in (a) showed discontinuities in the connection to scaffolds at both ends. In (b) the orientation of that scaffold has been reversed, resulting in a less disrupted binding energy plot. (Other scaffold-order changes outside of the shaded areas were made on the basis of other information, including marker and synteny analyses).

Figure 4 provides an example of the use of binding energy plots in chromosomal assembly for chromosome 2 (formerly D1b). The orientation of the darkened scaffold, scaffold_34, was provisionally reversed, on the assumption that a break in this gradient was unlikely to occur by chance precisely at the scaffold boundaries. The binding energy plot of the resulting assembly is shown in Figure 4(b).

**Figure 5:**
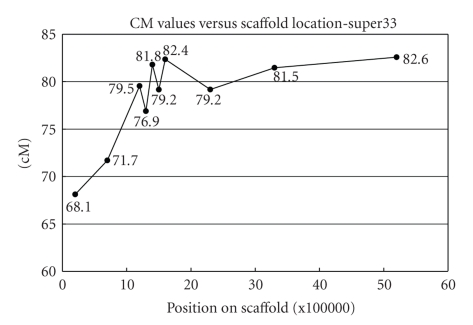
Position versus cM values for markers along super33.

When we further examined the marker data for that scaffold, we found suggestive evidence that the orientation shown in Figure 4(b) is correct. Figure 5 shows a plot of cM values versus physical location for scaffold_34 in the changed orientation. We note that the cM values of the first two markers, 68.1 and 71.7, are significantly less than the cM values of the last three markers, 79.2, 81.5, and 82.6. We also note that there is a flattening of the graph as we move in the pericentromeric region. This is what we expect as recombination events become rarer and marker resolution decreases. This provides additional evidence that the orientation of scaffold_34, shown in Figure 4(b), is indeed correct.

**Figure 6:**
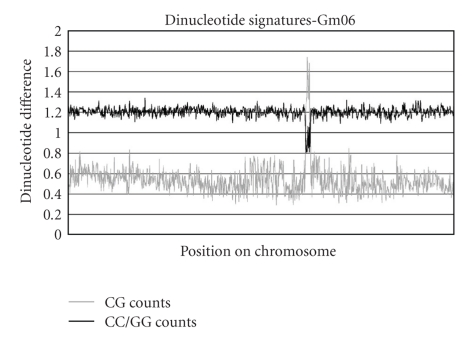
*ρ*_CG_* and *ρ*_CC/GG_* counts for 50 kb stretches along chromosome 6. The counts stay relatively stable until the centromere, where they differ significantly from the rest of the genome.

 After observing and utilizing the small-scale signals outlined above, we decided to further characterize the DNA in order to better understand the biological meaning behind the signals. For chromosome 6, we examined the individual ρ*_XY_ counts that were used to compute the dinucleotide differences shown in Figure 2. In the centromeric region, some dinucleotides increase in relative count while others decrease, leading to the aggregate differences shown in Figure 2. As an example of this, Figure 6 shows a plot of *ρ*_CG_* and *ρ*_CC/GG_* counts which illustrates the phenomenon. This analysis gives information about what dinucleotides frequencies differ along the chromosome but does not offer an underlying biological reason for those differences.

Using the etandem repeat finding software, part of the EMBOSS software suite [8], we identified many tandem repeats of dominant length 91 in that centromeric region. Those repeats have been characterized using FISH in previous studies [9]. We also searched for a representative 91-length repeat in chromosome 6 using wublast [10], retaining matches with at least 90% identity. Tandem arrays were then identified by counting hits that occurred within 910 base pairs (10x the repeat length) of each other. These arrays are found at exclusively one location–the centromere. *ρ*_CG_* and *ρ*_CC/GG_* counts, calculated directly from the 91 repeat, were 2.85 and 0.567, respectively, which differ significantly from the values of 0.540 and 1.21 for the entire soybean genome. Those CG and CC/GG count differences by themselves, when viewed in the context of how we determine δ* values, are enough to explain dinucleotide differences of ~0.2. This is approximately the height of the peak in Figure 2.

In attempting to explain the broad pericentromeric peaks in the binding energy plot of chromosome 2 (and all soybean chromosomes, data not shown), we analyzed the GC content of the euchromatic and heterochromatic regions as well as the GC content of a collection of LTR retrotransposons. The GC content of the LTR retrotransposons was 0.39, that of the euchromatic regions of chromosome 2 was 0.32, and that of the heterochromatic region of chromosome 2 was 0.37. When we removed the repeat content (LTRs and satellite repeats) from the heterochromatic region and recalculated, we saw a GC content of 0.33. This is enough to explain the broad peaks of Figure 4 and strongly suggests that it is the increased GC content of LTRs and repeats that lead to the patterns in binding energy.

We calculated similar binding energy and dinucleotide plots for chromosomes of grape, Arabidopsis, poplar, and rice to determine whether the patterns we observed in soybean were a general phenomenon or were specific to this species. Although we saw a few large-scale patterns along the chromosomes from those species, they appeared rarely and the patterns were in general more subdued than those seen in soybean (data not shown). Rice, poplar, and Arabidopsis showed relatively homogeneous peaks in the dinucleotide plots, with a noisy background consisting of narrow (~50–200 kb) peaks. The dinucleotide difference plot for the grape genome showed only minor, infrequent peaks. We are unsure whether the differences in the dinucleotide and binding energy plots were a result of differences between the genomes of those species or, rather, a difference in sequencing strategies used for the comparison genomes. The rice and poplar genomes were sequenced using clone-by-clone techniques and did not determine the sequence of all pericentromeric regions [11, 12]. The poplar genome was sequenced using a WGS strategy, but a larger proportion (~75 Mb) of the estimated genome was included in the pseudomolecule assemblies, and this excluded fraction was repeat dense [13].

## 3. Discussion

Marker data provided the bulk of the information necessary to order and orient the soybean WGS scaffolds, particularly in euchromatic regions. In addition, other tools were also useful across the genome, including FPC contigs, synteny plots, and gene- and retrotransposon-density data. However, in pericentromeric regions, the final assembly often required judgment calls after examining several pieces of inconclusive evidence. Thus, chromosomal assembly is not an exact science, particularly in centromeric and pericentromeric regions, where repeat arrays and a lack of marker resolution make higher-order assemblies problematic.

One property of a genome sequence, termed a dinucleotide signature, has been used to infer evolutionary history and structural organization of the genome. Dinucleotide signature data [14–17] was first used as a means to show that DNA was of opposite rather than similar polarity [18]. Since then it has been used to clarify phylogenetic relationships [14, 19–22] and provided evidence of horizontal transfer events between organisms [23–25]. In the latter application, it is the distinctive, relatively homogeneous signature of an organism's genome that allows putative foreign DNA to be identified. More recent work has suggested a correlation between changes in genomic signature and changes in DNA replication and repair machinery [26]. The evolutionary distances between DNA repair and recombination orthologs in a group of protobacteria correlated very highly with dinucleotide signature differences [26].

Until now, it has been generally accepted that, for any 50 kb stretch of a genome, ρ* for that segment varies little when compared to other 50 kb segments of the same genome. Differences between ρ* values for different organisms have been reported to be larger than differences between ρ* values for segments of the same organism [19, 27]. The soybean genome appears to challenge that conventional wisdom.

 Binding energy and dinucleotide difference plots provided additional information for the soybean assembly, but their utility was predicated on the existence of large-scale chromosomal patterns for both of these patterns. Broad patterns were not evident in poplar, Arabidopsis, or rice, but smaller-scale features were evident. Although we did not have the scaffold boundaries as an aide, this suggests that our technique could be used to guide some scaffold placements in other species. 

That the (C+G) content of euchromatic DNA (0.32) matched so closely the (C+G) content of heterochromatic DNA without LTR retrotransposons (0.33), coupled with the high (C+G) content of the LTR retrotransposons themselves (0.39), suggests that the broad peaks we see in the dinucleotide binding energy plot of chromosome 6 are connected to LTR retrotransposons. A strikingly high proportion (approximately 87%) of the LTR transposons in soybean is located in pericentromeric regions [28]. Remaining variability in (C+G) content dinucleotide signature in the pericentromere may be due to various features in the pericentromere, including ribosomal arrays and other genes (approximately 22% of genes predicted in the soybean genome occur within pericentromeric boundaries [1]). The *ρ*_CG_* and *ρ*_CC/GG_* values and localization of the length-91 repeats in the centromere suggest that the centromeric peaks are a result of the repeats.

We note that binding energy in the soybean data correlates very strongly with (C+G) content, defined as the ratio of total GC count over total nucleotide count. For the entire soybean genome the correlation was 0.999. Similarly, correlation in parts of the human genome between binding energy and (C+G) content was calculated to be 0.998 [29]. This suggests that in soybean (C+G) content and dinucleotide binding energy could be used interchangeably. We chose to use dinucleotide binding energy because we plan to compare this genome feature between soybean and other plant species.

## 4. Conclusions

We described a new technique for evaluating the placement of sequence scaffolds into linkage groups in areas of the chromosome where marker resolution is poor because of infrequent recombination events. The technique can highlight shifts in gradients and can identify possibly problematic scaffold placements; nevertheless, it should be used with other sources of information such as genetic and physical map data. There are other signals in DNA that could serve as additional pieces of information, such as nucleosome binding potential [29]. Many signals correlate strongly with (C+G) content, suggesting they would add little additional information because dinucleotide binding energy correlates so strongly with (C+G).

## 5. Methods

### 5.1. Genome Sequences

The soybean genomic sequence assemblies used in Figures 3 and 4 used scaffolds generated using the Arachne [10] assembler, constructed as part of the soybean genome consortium project [2]. Those draft assemblies led to the Glyma1.01 assembly, available at http://www.phytozome.net/soybean.php. The poplar (JGI, v1.0 (June 2004)) [13] and Arabidopsis (version TAIR 9.0) [11] genomes were downloaded from NCBI. The grape genome (assembly version 1, 2007) [30] was downloaded from the Grape Genome Browser (http://www.genoscope.cns.fr/externe/GenomeBrowser/Vitis/).

### 5.2. Dinucleotide Signature

The dinucleotide signature is based on the frequencies of the individual dinucleotides, normalized for the frequencies of the nucleotides. Let *f*_*X*_ be the frequency of nucleotide *X* in a genome and let *f*_*XY*_ be the frequency of dinucleotide *X* *Y*. We define

(1) $$\rho_{XY}=\frac{f_{XY}}{f_{X}f_{Y}}$$

as the signature of dinucleotide *X* *Y*, normalized for the percentages of the component nucleotides. Since genomic DNA is double stranded, we generalize

(2) $$\rho_{XY}^{*}=\sim \rho_{XY}=\frac{f_{XY}}{f_{X}f_{Y}}$$

to include the reverse compliment of a single-stranded sequence. Since there are 16 possible nucleotides, ρ* constitutes a vector signature for any given genome, consisting of the 16 individual dinucleotide signatures. We define ρ*(*f*) and ρ*(*g*) to be the vector signature of organisms *f* and *g* (or of regions *f* and *g* in the same genome). A coarse-grained measurement of the difference between the two organisms' signatures is thus defined by Karlin and Mrázek [21] as

(3) $$\begin{array}{cc} & \delta^{*}\left(f,g\right)=\;\left(\frac{1}{16}\right)\underset{XY}{\sum }\left|\rho_{XY}^{*}\left(f\right)-\rho_{XY}^{*}\left(g\right)\right|. \end{array}$$

  ρ* was calculated for the soybean genome as a whole, taking into account total nucleotide and dinucleotide counts for all chromosomes. Ns in the sequence were not included in either the nucleotide or dinucleotide counts. The vector was then compared with the vector from nonoverlapping 50 kb stretches of a chromosome, generating δ* values for all 50 kb stretches. This was done using custom perl scripts (available on request). For random sequences of DNA, the probability of observing values of *ρ*_XY_* greater than 1.23 or less than 0.78 was found to occur less than one in a thousand times [21, 31]. These values have been used to identify an over- and under-representation of a dinucleotide, respectively [14, 25, 31].

### 5.3. Dinucleotide Binding Energy

Thermodynamic stability of DNA has been used as a means of predicting coding regions and promoter locations of a genome [32–34]. The success of this method is largely dependant on the difference in (C+G) content between the regions of interest and the (C+G) content of the rest of the genome. Nearest neighbor (NN) free energy values can be used to calculate thermodynamic stability of DNA. Numerous studies have measured and exploited NN free energy values of the various dinucleotide pairs [35–38]. Table 1 gives a consensus for the free energy of binding of each of 16 pairs [38]. 

DNA binding energy was calculated using the Nearest Neighbor (NN) free energy values in Table 1. For a 50 kb segment of DNA, the total free energy of binding was calculated using the free energy for overlapping dinucleotides and dividing by the total number of dinucleotides. Ns in both the energy calculation and the nucleotide count were ignored. As with dinucleotide signature, the average binding energy was calculated, in 50 kb stretches, for all chromosomes. This was done using custom perl scripts (available on request).

### 5.4. Other

Tandem repeats were found using the etandem repeat finding software that is part of the EMBOSS software package [8]. Counts of (C+G) were calculated using custom perl scripts (available on request).

## Figures and Tables

**Table 1:** Free energy of binding at 37°C for all of the dinucleotide pairs [38]. The reverse-compliment pairs are shown together, resulting in 10 total unique pairs.

Dinucleotide Pair	ΔG° (kcal/mol)	Dinucleotide Pair	ΔG° (kcal/mol)
AA/TT	−1.00	CC/GG	−1.84
AC/GT	−1.44	CG	−2.17
AG/CT	−1.28	GA/TC	−1.30
AT	−0.88	GC/GC	−2.24
CA/TG	−1.45	TA	−0.58
